# No gadolinium K‐edge detected on the first clinical photon‐counting computed tomography scanner

**DOI:** 10.1002/acm2.14324

**Published:** 2024-03-12

**Authors:** Erik Baubeta, Eva Laurin Gadsböll, Leon Will, Fredrik Holmquist, Marie‐Louise Aurumskjöld

**Affiliations:** ^1^ Department of Imaging and Functional Medicine Skåne University Hospital Lund Sweden; ^2^ Department of Translational Medicine Diagnostic Radiology Lund University Malmö Sweden; ^3^ Department of Clinical Sciences Diagnostic Radiology Lund University Lund Sweden; ^4^ Medical Radiation Physics Department of Clinical Sciences Malmö Skåne University Hospital Lund University Lund Sweden

**Keywords:** computed tomography, EICT, energy integrated computed tomography, gadolinium contrast agent, iodine contrast agent, K‐edge, material decomposition, PCCT, photon counting computed tomography

## Abstract

**Purpose:**

This study aimed to elucidate whether gadolinium contrast in clinically relevant doses can be used with photon‐counting computed tomography (PCCT) as an alternative contrast agent in clinical applications.

**Material/methods:**

A CTDI phantom with 3D printed rods filled with different concentrations of gadolinium and iodine contrast was scanned in a PCCT and an energy‐integrated computed tomography (EICT). Attenuation values at different monoenergetic steps were extracted for each contrast concentration.

**Results:**

For PCCT, gadolinium reached an attenuation >100 HU (103 HU) at 40 keV with a concentration 5 mmol/L whereas the same level was reached at 50 keV (118 HU) for 10 mmol/L and 90 keV (114 HU) for 25 mmol/L. For iodine, the same level of attenuation was reached at 100 keV (106 HU) with a concentration 8.75 mg I/mL.

For EICT the lowest gadolinium contrast concentration needed to reach >100 HU (108 HU) was 10 mmol/L at 50 keV. For 25 mmol/L 100 HU was reached at 100 keV. For iodine contrast 108 HU was reached at 110 keV for 8.75 mg I/mL.

**Conclusion:**

No K‐edge potential or difference in attenuation curves between iodine and gadolinium contrast is detected on the first clinical available PCCT. Clinically relevant attenuation levels were barely achieved in this setting with gadolinium concentrations approved for human use. The results of this study suggest that, given current scanning technology, gadolinium is not a clinically useful contrast agent for computed tomography because no K‐edge was detected.

## 1 INTRODUCTION

Photon counting computed tomography (PCCT) is a relatively new imaging technique that offers significant advantages over energy‐integrated computed tomography (EICT). Unlike traditional CT scans, which measure X‐ray photons converted into light, PCCT counts individual photons, allowing for more accurate detection of the energy spectrum and potentially reducing the amount of radiation and contrast agent required.^1^, ^2^ This reduction of electron noise makes it possible to increase contrast‐to‐noise ratio (CNR) and signal‐to‐noise ratio (SNR) but also enable imaging with reduced radiation dose. Further, the technique makes it possible to obtain images with finer details and higher resolution.^3^, ^4^

One area in which PCCT has the potential to change how CT is used is the ability to differentiate between different materials and thereby the use of multiple contrast media. Alternative contrast media with different K‐edge within the spectrum of PCCT and its potential has been suggested as a future technique to evolve diagnostics.^5^, ^6^ In this field numerous phantom and animal studies have been performed in preclinical PCCT equipment showing promising results.^7^, ^8^ Some benefits of using multiple contrast agents in clinical practice could be higher diagnostic accuracy in differentiating uptake and distribution of the contrast agents.^9^ Another benefit would be faster examination times and reduced radiation dose as differentiation of contrast can be made in post‐processing after one single examination.

Iodine and gadolinium‐based contrast agents are widely used and approved for use in humans. Iodine is primarily used in conventional X‐ray‐based imaging techniques such as X‐ray, angiography and CT, whereas gadolinium is primarily used in magnetic resonance imaging (MRI). However, both these contrast agents can be used in conventional X‐ray examinations but gadolinium concentrations need to be higher to be detected on conventional X‐ray.^10^ This, however, is a potential disadvantage that could be overcome with the PCCT's higher sensitivity to contrast media and the ability to change monoenergetic steps.^1^, ^2^ This ability to detect different materials K‐edge and optimize is what makes this technique interesting. The K‐edge is a powerful technique for studying the structural, electronic, and magnetic properties of materials by analyzing their X‐ray absorption spectra. It provides information about the chemical composition, electronic structure, and local symmetry of the atoms surrounding the absorbing element.^2^ Iodine with its K‐edge at 33.2 keV and gadolinium with K‐edge at 50.2 keV could be placed in different bins in the PCCT and thereby separated from each other at different monoenergetic levels. Further, using the increased signal at gadoliniums K‐edge could reduce the doses needed to obtain clinically relevant images. This idea has previously been studied using dual‐energy CT with poor results when using clinical doses of gadolinium.^11^ The advantages of PCCT mentioned above could potentially change this and create possibilities for the use of gadolinium contrast in CT applications.

In 2021 the first commercially available PCCT scanner approved for clinical use (NAEOTOM Alpha, Siemens Healthcare, Erlangen, Germany) was introduced. The potential is large in the field of clinical applications where the use of multiple contrast agents could be helpful. As a first step, the concept needs to be proven in a clinically approved PCCT scanner and relevant contrast levels need to be determined. Therefore, this phantom study aims to evaluate the response of the PCCT scanner to different concentrations of gadolinium to evaluate the clinical feasibility of K‐edge imaging.

## 2 METHODS

### 2.1 The phantom

**FIGURE 1:**
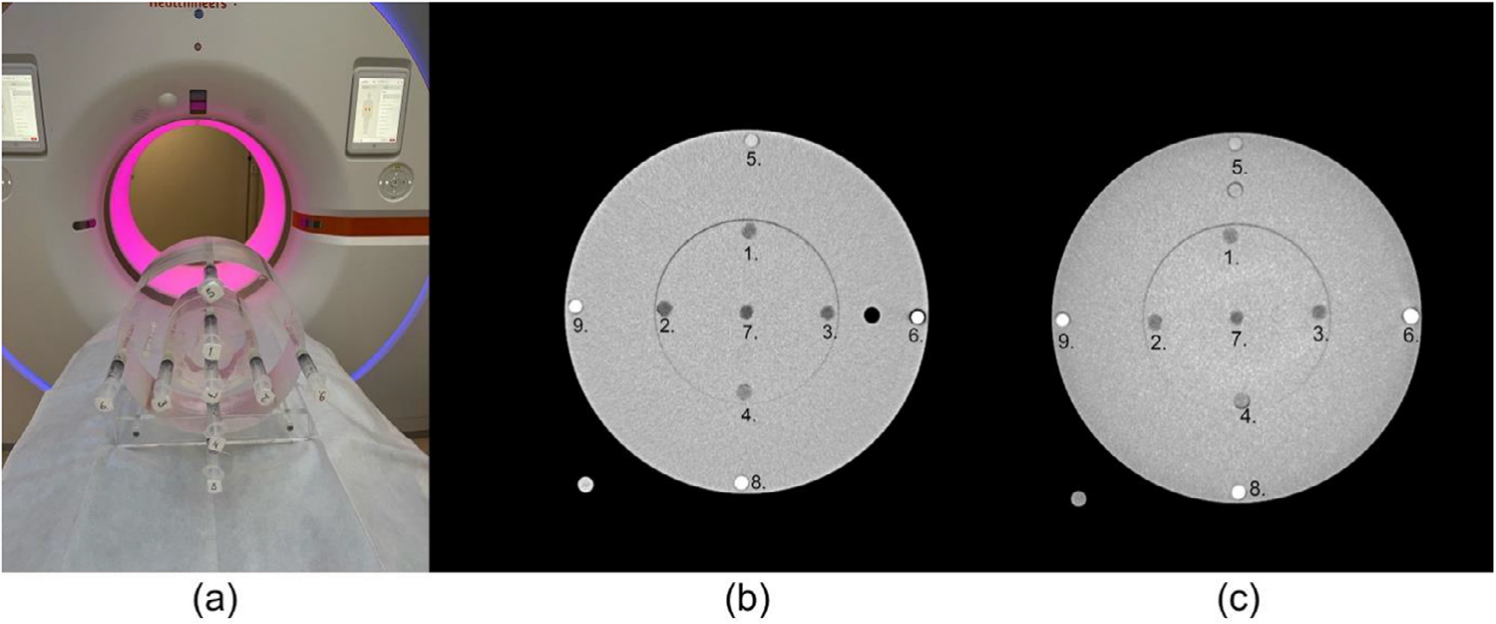
(a) The phantom in the PCCT and the 3D printed rods connected to syringes with iodine and gadolinium solutions. The corresponding (b) PCCT image, and (c) EICT image.

A 32 cm CTDI phantom (length 19 cm; material polymethyl methacrylate (PMMA); density 1.19 g/cm^3^; body diameter 32 cm cylinder with a hole diameter 1.3 cm) with eight 3D printed insert rods filled with iodine and gadolinium solutions was used (Figure 1). Each rod had a diameter of 1.3 cm, a length of 18 cm, wall thickness 0.8 mm and made of clear material (Formlabs) and was printed using stereolithography (SLA). The rods were equipped with Luer‐lock connections in both ends to be able to be filled without residual air to minimize risk of artifacts. The contrast agents used in this study were gadoteric acid (Clariscan 279.3 mg/mL; 0.5 mmol/mL, GE Healthcare) and Iohexol (Omnipaque, 350 mg I/mL, GE Healthcare). The different concentrations of iodine and gadolinium are presented in Table 1. The gadolinium concentrations were chosen with the aim of representing typical blood concentrations in humans when using clinically approved doses of gadolinium contrast.^12^, ^13^, ^14^, ^15^ During the scan we filled one rod with pure gadoteric acid (labeled “6” in Figure 1; Clariscan 279.3 mg/mL; 0.5 mmol/mL) as reference. The results from this rod were excluded from the analysis because its attenuation was significantly higher than the other samples, thereby disrupting the interpretation of the remaining clinically relevant samples. As reference, one rod was filled with salt water solution with 0.9% NaCl by weight. All rods were imaged simultaneously in the phantom.

**TABLE 1:** Gadolinium and iodine concentrations in the 3D printed rods.

Rod number	Contrast agent	Concentration in rods		
3	Gadoteric acid	0.70 mg/mL	1.25 mmol/L	
2	Gadoteric acid	1.40 mg/mL	2.5 mmol/L	
1	Gadoteric acid	2.79 mg/mL	5 mmol/L	Equivalent to portovenous phase scan^13^
4	Gadoteric acid	5.59 mg/mL	10 mmol/L	Equivalent to arterial phase scan^12^, ^13^
5	Gadoteric acid	13.97 mg/mL	25 mmol/L	
7	Salt water solution	0.9% NaCl by weight		Used for reference
8	Iohexol	17.5 mg/mL		Used for reference
9	Iohexol	8.75 mg/mL		Used for reference

*Note*: Rod numbers as indicated in Figure 1.

### 2.2 Scanning

The phantom was scanned in two different scanners: a PCCT (NAEOTOM Alpha, Siemens Healthcare, Erlangen, Germany) and EICT (Somatom Definition Flash, Siemens Healthcare, Erlangen Germany). To optimize image quality and minimize risk of scanning parameters resulting in erroneous readouts, a scan with a high radiation dose with a volume CT dose index (CTDIvol) 21 was used on both scanners. The CT scanning parameters used in this study are shown in Table 2.

**TABLE 2:** A summary of the scan parameters used.

	PCCT	EICT	
Scan parameters	High radiation dose	High radiation dose	DE
Tube voltage (kV)	140	100	80/Sn140
Tube current (mAs)	266	510	305/118
Rotation time (s)	0.25	0.5	0.33
Reconstruction smoothing kernel	Br44	Br38f	Br38f
Reconstruction algorithm	QIR, level 4	Safir, level 2	Safir, level 3
CTDIvol	21	21	10.1

Abbreviations: DE, dual energy; EICT, energy integrated computed tomography; PCCT,  photon counting computed tomography; QIR, quantum iterative reconstruction; Safir, sinogram affirmed iterative reconstruction.

### 2.3 Image evaluation

The different keV steps were extracted from the raw file (SPP) and this post processing was made using the provided software (Syngovia Siemens, Erlangen, Germany). All measurements were made on the axial slides. In a dedicated workstation a ROI was placed in the location of each sample. ROIs were placed centrally in the rod aiming at as large ROIs as possible. The ROIs had a mean size of 65 mm^2^ (±5 mm^2^). For reference, a ROI was placed in the phantom located between the rods where the phantom was homogenous with no visible artifacts. The attenuation (Hounsfield units, HU) was extracted in each monoenergetic step (40 keV – 140 keV in 10 keV steps). A cut‐off value of 100 HU was assumed significant for clinical applications to be able to identify contrast enhancement. This cut‐off value was chosen based on unenhanced solid organs and a potentially detectable difference in contrast enhancement.^16^, ^17^

## 3 RESULTS

All levels of gadolinium and iodine concentrations gave an increase in attenuation compared to pure NaCl and the phantom (Table 3). The increase was applicable both to PCCT and EICT.

**TABLE 3:** Attenuation in the 3D printed rods with different concentrations of iodine and gadolinium.

		Mean Hounsfield unit ± Standard deviation											
		40 keV		50 keV		60 keV		70 keV		80 keV		90 keV	
Rod #	Phantom region	EICT	PCCT	EICT	PCCT	EICT	PCCT	EICT	PCCT	EICT	PCCT	EICT	PCCT
5	Gd 25 mmol/L	–	470 ± 16	273 ± 72	321 ± 11	202 ± 42	230 ± 8	159 ± 26	174 ± 6	131 ± 22	138 ± 7	113 ± 23	114 ± 7
4	Gd 10 mmol/L	–	159 ± 22	108 ± 64	118 ± 16	85 ± 32	92 ± 12	70 ± 21	74 ± 11	61 ± 24	67 ± 12	55 ± 30	60 ± 13
1	Gd 5 mmol/L	–	103 ± 17	86 ± 88	79 ± 14	65 ± 51	63 ± 12	52 ± 33	54 ± 10	44 ± 28	48 ± 9	38 ± 30	44 ± 8
2	Gd 2.5 mmol/L	–	45 ± 21	77 ± 72	39 ± 17	51 ± 43	35 ± 13	36 ± 34	31 ± 10	26 ± 35	28 ± 10	19 ± 39	27 ± 10
3	Gd 1.25 mmol/L	–	39 ± 21	16 ± 51	36 ± 17	21 ± 31	34 ± 15	25 ± 26	33 ± 12	27 ± 28	33 ± 11	29 ± 31	33 ± 12
8	I 17.5 mg/mL	–	1735 ± 26	975 ± 112	1119 ± 20	684 ± 68	746 ± 17	504 ± 44	516 ± 12	389 ± 32	369 ± 9	312 ± 28	271 ± 8
9	I 8.75 mg/mL	–	945 ± 23	539 ± 49	607 ± 15	372 ± 29	402 ± 10	270 ± 20	277 ± 7	204 ± 16	197 ± 8	160 ± 17	143 ± 9
7	0.9% NaCl	–	39 ± 21	−18 ± 87	29 ± 18	−5 ± 51	21 ± 15	3 ± 38	14 ± 10	8 ± 37	11 ± 10	11 ± 40	10 ± 10
–	Phantom	–	63 ± 23	78 ± 98	97 ± 18	104 ± 55	118 ± 14	120 ± 37	130 ± 11	130 ± 36	139 ± 11	137 ± 40	144 ± 11

		Mean Hounsfield unit ± Standard deviation									
		100 keV		110 keV		120 keV		130 keV		140 keV	
Rod #	Phantom region	EICT	PCCT	EICT	PCCT	EICT	PCCT	EICT	PCCT	EICT	PCCT
5	Gd 25 mmol/L	100 ± 25	97 ± 7	91 ± 28	85 ± 7	84 ± 30	76 ± 8	80 ± 31	70 ± 7	76 ± 33	65 ± 8
4	Gd 10 mmol/L	51 ± 35	56 ± 13	48 ± 39	53 ± 13	46 ± 42	51 ± 13	44 ± 44	49 ± 13	43 ± 46	48 ± 13
1	Gd 5 mmol/L	34 ± 32	42 ± 8	32 ± 35	40 ± 9	29 ± 38	38 ± 9	28 ± 40	37 ± 9	27 ± 41	36 ± 9
2	Gd 2.5 mmol/L	14 ± 43	26 ± 10	11 ± 45	26 ± 10	9 ± 48	25 ± 10	7 ± 50	25 ± 10	6 ± 51	25 ± 10
3	Gd 1.25 mmol/L	30 ± 34	33 ± 12	30 ± 36	32 ± 12	31 ± 38	32 ± 13	31 ± 39	32 ± 13	32 ± 43	32 ± 13
8	I 17.5 mg/mL	259 ± 29	203 ± 8	222 ± 30	154 ± 8	194 ± 32	119 ± 9	174 ± 34	92 ± 9	158 ± 35	72 ± 9
9	I 8.75 mg/mL	130 ± 18	106 ± 9	108 ± 19	80 ± 9	92 ± 20	60 ± 9	81 ± 21	46 ± 10	72 ± 22	35 ± 10
7	0.9% NaCl	14 ± 45	10 ± 10	16 ± 48	9 ± 10	17 ± 51	9 ± 10	18 ± 53	8 ± 10	18 ± 55	8 ± 10
–	Phantom	142 ± 45	148 ± 11	145 ± 49	150 ± 11	148 ± 53	152 ± 11	150 ± 55	154 ± 11	151 ± 57	155 ± 11

*Note*: Rod numbers as indicated in Figure 1.

Abbreviations: EICT, energy integrated computed tomography; PCCT, photon counting computed tomography.

**FIGURE 2:**
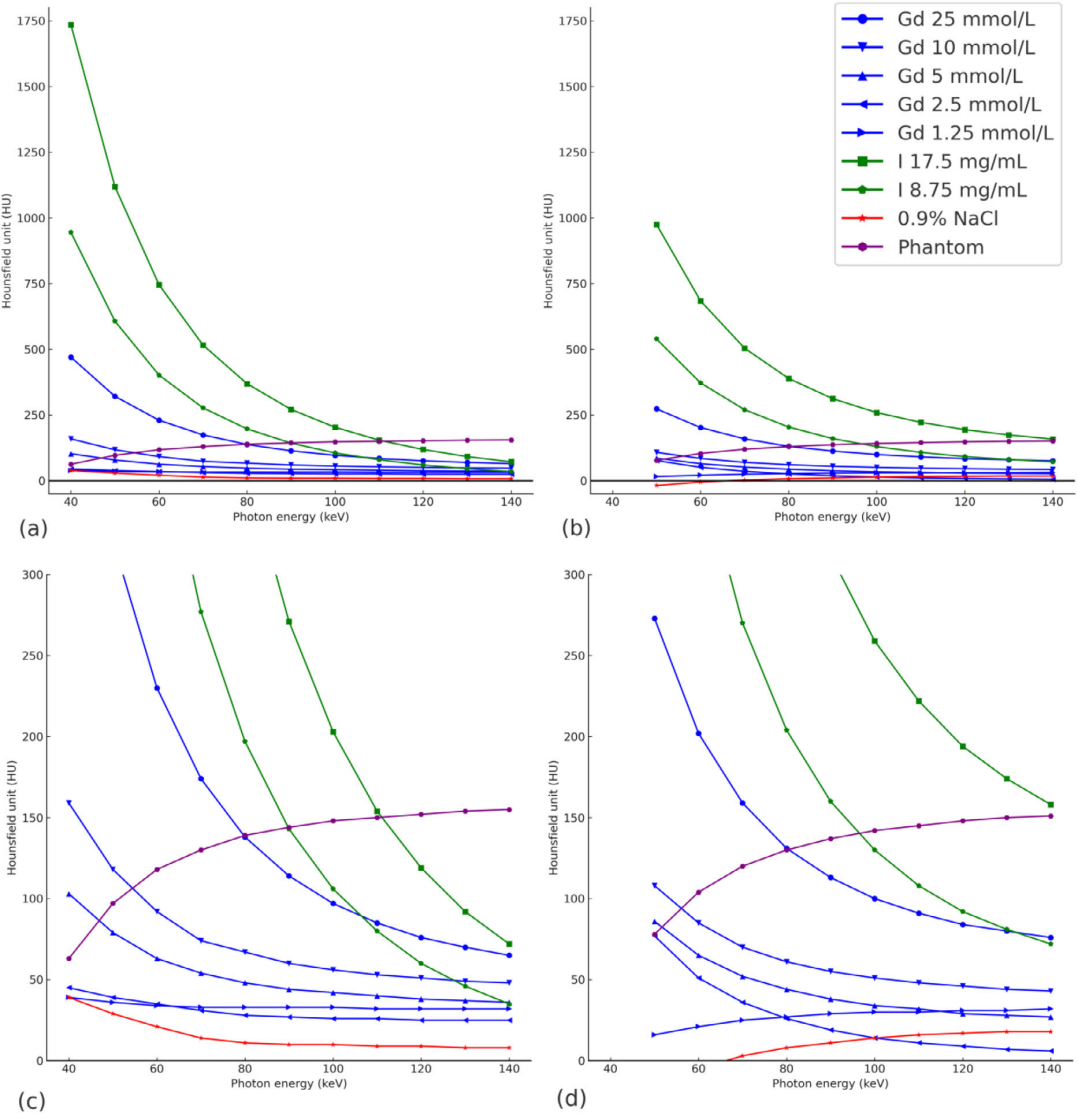
Attenuation for contrast agents at various concentrations and keV steps. (a) PCCT and (b) EICT graphs. A section of attenuation between 0 and 300 HU is showed for (c) PCCT and (d) EICT.

Attenuation decreased with increasing photon energy, but HU tends to approach an asymptotic increase with increasing energy for the PCCT, especially with lower concentrations of gadolinium (Figure 2). For PCCT, gadolinium reached an attenuation > 100 HU (103 HU) at 40 keV with a concentration 5 mmol/L whereas the same level was reached at 50 keV (118 HU) for 10 mmol/L and 90 keV (114 HU) for 25 mmol/L. For iodine, the same level of attenuation was reached at 100 keV (106 HU) with a concentration 8.75 mg I/mL.

For EICT it was not possible to extract data from 40 keV because of high noise ratios. The lowest gadolinium contrast concentration needed to reach >100 HU (108 HU) was therefore 10 mmol/L at 50 keV. For 25 mmol/L 100 HU was reached at 100 keV. For iodine contrast 108 HU was reached at 110 keV for 8.75 mg I/mL.

## 4 DISCUSSION

In this phantom study, performed at the first clinically available PCCT, we did not see a discrimination between gadolinium and iodine‐based contrast agents. No K‐edge was visualized and therefore no relevant gadolinium concentrations for use in clinical practice was able to be established.

When we measured attenuation of gadolinium and iodine at different concentrations at different monoenergetic steps we could not see a difference between the curves from iodine and gadolinium except for attenuation. The curves evolved the same. Since our monoenergetic steps reached over the theoretical K‐edge of gadolinium (50.2 keV) we would have anticipated an increase of attenuation in gadolinium at 50 keV. Instead, both curves had an exponential linear distribution expected to that of iodine. As suggested in previous studies on alternative contrast agents we would expect a steep increase in the attenuation curve of gadolinium at 50 keV.^5^ Since this is not what we expected and not what was seen in studies using preclinical PCCT,^7^, ^8^ one hypothesis is that the clinical PCCT assumes increased attenuation is iodine, since this is what normally is used in clinical practice. This theory is partially confirmed by statements from the manufacturer.^18^

Prior studies performed in dual‐energy CT with gadolinium‐based contrast agents failed to measure an increased attenuation in liver parenchyma and detect the K‐edge, probably because of too low contrast concentrations.^11^ These findings are confirmed by our comparison with the EICT. However, in PCCT with the higher sensitivity of photon counting and the use of optimizing attenuation based on K‐edge, it would theoretically be possible to detect lower concentrations than in conventional CT technique. In our study, we tried to optimize this by testing both clinically relevant and much higher contrast concentrations to overcome these concentration issues. Despite this, there is a discrepancy between what the expected finding would be and the actual outcome of the testing. On the other hand, one interesting finding in this study is that the increased sensitivity of the PCCT resulted in higher attenuation values at the same contrast concentration levels. Even though we did not observe the peak at the K‐edge, the higher sensitivity of PCCT made the attenuation values at clinically acceptable doses of gadolinium approach clinically relevant levels. This is an interesting finding that supports the use of gadolinium as a contrast agent in PCCT when the K‐edge potential can be utilized. In this study, where we measured actual attenuation levels, no subtraction was performed. However, in a clinical context, where K‐edge images are to be assessed visually, K‐edge images would consist of subtracted images, further improving the visualization of the contrast agent.^6^, ^9^, ^19^ The attenuation of the phantom increased with photon energy for both PCCT and EICT. One explanation for this could be the difference in the material PMMA (mass attenuation coefficient) compared to water.

If we assume a clinically relevant attenuation value would be around 100 HU to be able to differentiate contrast enhancement from the background soft tissue attenuation the lowest concentrations of gadolinium would not be sufficient for clinical use. Both the gadoteric acid 1.25 mmol/L and 2.5 mmol/L concentrations did not reach over 100 HU in any of the monoenergetic steps. However, the 5 mmol/L concentration (equivalent to port venous phase enhancement in human^13^) reached 103 HU at 40 keV and could potentially be used in clinical applications. If we had been able to detect the K‐edge we would theoretically have seen an increase in attenuation around this level (theoretically around 50 keV) which could have pushed even the lower concentrations over 100 HU or further increased the attenuation of the contrast enhancement for an increased clinical relevance. In previous animal and phantom studies contrast doses have not been an issue since iodine concentrations are not of concern in these specific situations but have shown promising results for the potential of the technique.^7^, ^8^, ^19^

To our knowledge, this is the first time this comparison has been made in a clinically available PCCT and raises concerns on how the algorithm of the PCCT handles the different contrast agents and what assumptions are made. However, the fact that clinically relevant attenuation levels were achieved even without the K‐edge amplification is promising for the future use of gadolinium contrast agents in PCCT applications.

A weakness in this study is the use of the standard hardware and software provided with the first generation PCCT for clinical use. This software might not unlock the full potential of the hardware. However, the aim of this study was to elucidate whether the machine could be used practically in the ways it could theoretically be used, and to this question we have a result.

The strength of the study is the use of a standardized phantom with 3D printed rods where all contrast concentrations could be scanned at the same time with the same hard‐ and software parameters. Furthermore, we compared the results from the PCCT with a dual energy EICT using the same phantom and contrast solutions.

## 5 CONCLUSION

No K‐edge potential or difference in attenuation curves between iodine and gadolinium contrast is detected on the first clinical available PCCT. Clinically relevant attenuation levels were barely achieved in this setting with gadolinium concentrations approved for human use. The results of this study suggest that, given current scanning technology, gadolinium is not a clinically useful contrast agent for computed tomography because no K‐edge was detected.

## AUTHOR CONTRIBUTIONS

EB—Conceptualisation. Sample preparation and collection. Writing and drafting the manuscript. ELG—Sample preparation and collection. Drafting, finalizing and approval of the manuscript. LW—Contributed to sample preparation. Drafting, finalizing and approval of the manuscript. FH—Sample preparation. Drafting manuscript and final approval. MA—Conceptualisation. Sample preparation and collection. Drafting, finalizing and approval of the manuscript.

## CONFLICT OF INTEREST STATEMENT

The authors declare no conflicts of interest.
